# Genetic mapping of morpho-physiological traits involved during reproductive stage drought tolerance in rice

**DOI:** 10.1371/journal.pone.0214979

**Published:** 2019-12-17

**Authors:** Saumya Ranjan Barik, Elssa Pandit, Sharat Kumar Pradhan, Shakti Prakash Mohanty, Trilochan Mohapatra

**Affiliations:** 1 ICAR-National Rice Research Institute, Cuttack, Odisha, India; 2 Indian Council of Agricultural Research, New Delhi, India; North Dakota State University, UNITED STATES

## Abstract

Reproductive stage drought stress is an important yield reducing factor in rainfed rice. Genetic mapping of morpho-physiological traits under the stress will help to develop cultivars suitable for drought prone environments through marker-assisted breeding (MAB). Though various yield QTLs under reproductive stage drought tolerance are available for MAB, but no robust markers controlling different morho-physiological traits are available for this stress tolerance. QTLs linked to morpho-physiological traits under drought stress were mapped by evaluating 190 F_7_ recombinant inbred lines (RIL) using bulk segregant analysis (BSA) strategy. Wide variations were observed in the RILs for eleven morpho-physiological traits involved during the stress. A total of 401 SSR primers were surveyed for parental polymorphism of which 77 were detected to be polymorphic. Inclusive composite interval mapping detected a total of five consistent QTLs controlling leaf rolling (*qLR*_*9*.1_), leaf drying (*qLD*_*9*.1_), harvest index (*qHI*_*9*.1_), spikelet fertility (*qSF*_*9*.1_) and relative water content (*qRWC*_*9*.1_) under reproductive stage drought stress. Another two non-allelic QTLs controlling leaf rolling (*qLR*_*8*.1_) and leaf drying (*qLD*_*12*.1_) were also detected to be linked and found to control the two traits. QTL controlling leaf rolling, *qLR8*.*1* was validated in this mapping population and may be useful in MAB programs. Out of these five consistent QTLs, four (*qLR*_*9*.1,_ *qLD*_*9*.1,_ *qHI*_*9*.1_ and *qRWC*_*9*.1_) were detected to be novel QTLs and useful for MAB for improvement of reproductive stage drought tolerance in rice.

## Introduction

Rice is the staple food for more than half of the global population. This crop provides livelihood security to the majority of rural people in Asia and South-east Asia. Globally, the crop is cultivated in about 160.8 million hectares with annual production of more than 725.5 million tons of paddy [1]. The food production need to be increased even from the drought-prone areas with an increase of 40% from the difficult ecosystem to meet the targeted food production by 2025 [2]. Drought is the main abiotic constraint that reduces the rice yield greatly in rainfed rice environment [3,4]. Although rice production is increasing by 2.8% annually, the damage due to biotic and abiotic factors account for a heavy loss of global production [5]. In Asia, about 34 million hectares of rainfed lowland and 8 million hectare of upland rice are affected by frequent drought stress [6]. In recent years, the time and frequency of drought spells are increasing in the India due to the effects of climate change. The water deficit and frequency of dry spell will further increase in the coming years [7,8]. The drought tolerance mechanism is complex and controlled by many quantitative trait loci (QTLs) [9]. It affects the crop at both vegetative and reproductive growth stages of the crop. Identification of traits involved during drought stress tolerance and the candidate genes and QTLs controlling those traits will help the breeders in drought improvement program.

Photosynthetic capacities of leaves and water availability to the root zones are very important factors that reduces yield in susceptible rice genotypes under drought stress condition in rice [10]. The stress may reduce many morphological and physiological changes including grain yield. The stress reduces CO2 assimilation rates, photosynthetic pigments, water use efficiency, stomatal conductance, sucrose biosynthetic enzymes and translocation of assimilates, phloem loading and starch- activities resulting in low grain yield [11]. Leaf rolling and leaf drying are good criteria of screening drought tolerance during vegetative stage in rice [12–14]. Drought tolerance may be manifested through drought escape, drought avoidance, drought tolerance and drought recovery capacity. Several genes/QTLs governing these four drought tolerance mechanism have been reported earlier [6,15–29]. Rice genotypes showing delay in leaf rolling under drought and quick recovery from stress condition are the traits required in rice drought breeding program [30]. Reproductive stage drought stress is very harsh to rice crop as it reduces grain yield drastically due to more sterile grains in the panicles [14,31–35]. The stress reduces grain weight, size and spikelet fertility and finally resulting low yield [36–37]. A drastic decrease in grain yield and its component traits is usually seen due to the reproductive stage drought stress at a soil moisture level reaching to -60kPA or more. Reproductive stage is regarded as the most crucial stage of drought stress in compare to vegetative stage drought [38–39]. The responses to drought in the vegetative stage and reproductive stage are much different [40]. Several experiments have been reported previously for morpho-physiological responses under reproductive stage drought stress [41–26,44,45,27,46–50].

Several QTLs and genes on different chromosomes associated with different traits directly or indirectly for drought tolerance have been identified and mapped earlier from several studies by using different mapping populations [28,51–56]. However, more number of robust markers for gene (s)/QTLs controlling various traits for the stress tolerance need to be increased to speed up the slow pace of drought tolerance improvement in rice. Though the various yield QTLs reported under reproductive stage drought tolerance [29,57–62] are reported for marker-assisted breeding, but stronger markers need to be identified for several morho-physiological traits under stress tolerance. Results of this mapping study may be useful. Therefore, morpho-physiological traits linked to QTLs under drought stress were mapped by phenotyping 190 F_7_ recombinant inbred lines (RILs) of CR 143-2-2 / Krishnahamsa with the microsatellite polymorphic markers using bulk segregant analysis approach.

## Materials and methods

### Plant materials

One hundred ninety RILs in F_7_ generation developed from the cross of contrasting parents for drought tolerance, CR 143-2-2 and Krishnahamsa were used for the mapping study. CR 143-2-2, a drought tolerant donor line possessing both vegetative and reproductive stage drought tolerance and the contrasting parent, Krishnahamsa (susceptible variety for drought) were chosen for development of mapping population. Single seed descent (SSD) method was followed in each generation (F_2_-F_7)_ for development of the recombinant inbred lines. RILs were raised under control facility in the rain-out shelter (RoS) of ICAR-National Rice Research Institute (NRRI) during two consecutive years *i*.*e*., wet season, 2014 and 2015. CR 143-2-2 is an upland genotype developed by NRRI, Cuttack used as the tolerant parent while Krishnahamsa (DRR Dhan20) developed by Indian Institute of Rice Research, Hyderabad was taken as susceptible parent for drought tolerance mapping study.

### Phenotyping of physiological traits under reproductive stage drought stress

The seeds of RILs were direct seeded in alpha lattice design and replicated twice in the rain out shelter. The seedlings were maintained in normal condition by irrigation up to vegetative stage of the crop. All the recombinant lines along with their parents were arranged in six blocks constituting 34 lines per block with spacing of 10 x 15 cm. Each row accommodated 25 hills per each RIL. Ten hills sample data from each RIL and replication were taken up for evaluation and data analysis. The recommended fertilizer dose and need based plant measures were followed to maintain a good crop. Drought stress was applied at early PI (primordium initiation) stage. The drought stress was maintained continuously up to -50kPA throughout the reproductive stage in the experimental field. Eleven morpho-physiological traits *viz*., plant height, leaf rolling, leaf drying, panicle length, percentage of panicle emergence, harvest index, 1000-grain weight, percentage of spikelet fertility, relative water content, cell membrane stability and grain yield were estimated under the stress condition.

Data for plant height of each recombinant lines and parents were recorded during growth stage 7. Other pre-harvested data *viz*., leaf rolling, leaf drying, panicle length and percentage of panicle emergence were recorded during growth stage of 6–9 [63]. Post-harvest data namely harvest index, 1000-grain weight, spikelet fertility (%) and grain yield/plant were recorded from 10 hills during growth stage 9 of the crop. The samples for relative water content and cell membrane stability were collected from the field at mid-day during 7–8 crop growth stage. Estimation of relative water content was done as per the method of [64]. The samples for cell membrane stability were collected at mid-day and estimation of the trait was performed following the procedure of [65].

### Genotyping

#### DNA extraction

Twenty day old leaf samples were collected from the population aseptically for genomic DNA extraction. Leaves were homogenized with the help of liquid nitrogen in mortar and pestle collected in 2ml micro centrifugation tube. Pre warmed (65°C) Cetyltrimethyl ammonium bromide (CTAB) extraction buffer (2% CTAB, 100mM Tris pH 8, 20mM Ethylene diamine tetra acetate (EDTA) pH 8, 1.3M NaCl) was added to the sample followed by phenol chloroform isoamyl alcohol extraction, RNase treatment and ethanol precipitation [66]. The quality and quantity of final extracted DNA was verified using λ-DNA on 1% agarose gel. Also, DNA quantification and purity was further checked by measuring the OD at 260 and 280nm using a UV visible spectrophotometer. The samples were diluted accordingly for their uniformity to approximately 30ng/μl.

#### Polymerase chain reaction (PCR)

The polymerase chain reaction was performed by taking 20μl aliquot in a programmable thermal cycler (Veriti, Applied-Biosystems) using SSR primers (Table 1; Table 2). The PCR reaction mixture included 30ng genomic DNA, 10mM dNTPs, 2mM MgCl_2_, 50mM KCl, 1.5 mM Tris HCl (pH 8.75), 1U Taq polymerase and 10μM each of forward and reverse primers. The thermal profile starts with initial denaturation at 94°C for 4 min continued to 35 cycles of denaturation at 94°C for 30 sec, primer annealing 55°C for 1 min, extension 72°C for 1.30 min and final extension at 72°C for 10 min. After completion of amplification, PCR products were kept in a -20°C deep freeze. An aliquot of 10 μl PCR amplification products were loaded in an agarose gel of 3.5% containing 0.08 μg/ml of ethidium bromide for electrophoresis. The electrophoresis was carried out at 80 volts (2.5 V/cm) in 1X TBE (pH 8.0). Sizes of amplicons were determined by using 50bp DNA ladder. The photographs of banding pattern were recorded using gel documentation unit (Syngene G Box).

**Table 1:** Microsatellite markers obtained through the polymorphic analysis between CR143-2-2 and Krishnahamsa.

Chromosome	No. of markers analyzed	Total No. and names of the parental polymorphic markers obtained		Total No. and names of the bulked polymorphic markers used	
1	50	10	RM6703, RM3825, RM488, RM259, RM5, RM12091, RM8085, RM495, RM5443, RM1003	3	RM495, RM6703, RM3825
2	50	11	RM324, RM263, RM327, RM530, RM262, RM3549, RM279, OSR17, RM250, RM 341, RM13600	3	RM327, RM341, RM263
3	42	12	RM523, RM231, RM7332, RM517, RM411, RM135, RM85, RM22, RM16030, RM15780, RM104, RM571	2	RM22, RM517
4	26	--	--	--	--
5	8	--	--	--	--
6	30	4	RM3, RM276, RM527, RM528	2	RM527, RM3
7	14	1	MGR4499	--	--
8	22	6	RM256, RM337, RM210, RM25, RM342A, RM 72	2	RM337, RM72
9	38	7	RM464, RM215, RM219, RM316, RM257, RM242, RM213	2	RM316, RM257
10	26	6	RM216, RM228, RM311, RM271, RM171, RM484	3	RM271, RM171, RM484
11	10	1	RM21	--	--
12	84	19	RM28199, RM28089, RM511, RM28166, RM1261, RM28048, RM28059, RM28064, RM28067, RM28070, RM28079, RM28082, RM28083, RM28088, RM28090, RM519, RM313, RM309, RM20A	4	RM20A, RM511, RM309, RM519
Total	401	77		21	

**Table 2:** Details of SSR markers detected in the genotyping of RILs using the polymorphic primers used in QTL mapping.

Marker name	Chrom#	position	Forward primer	Reverse primer	Repeat motif	Annl temp
RM495	1	2.8	AATCCAAGGTGCAGAGATGG	CAACGATGACGAACACAACC	(CTG)7	55°C
RM6703	1	139.1	CAGCAAACCAAACCAAGCC	GCGAGGAGGAGGAGAAAAAG	(TAC)12	55°C
RM3825	1	143.7	AAAGCCCCCAAAAGCAGTAC	GTGAAACTCTGGGGTGTTCG	(GA)21	55°C
RM327	2	72.6	CTACTCCTCTGTCCCTCCTCTC	CCAGCTAGACACAATCGAGC	(CAT)11(CTT)5	55°C
RM341	2	94.4	CAAGAAACCTCAATCCGAGC	CTCCTCCCGATCCCAATC	(CTT)20	55°C
RM263	2	127.5	CCCAGGCTAGCTCATGAACC	GCTACGTTTGAGCTACCACG	(CT)34	55°C
RM22	3	7.2	GGTTTGGGAGCCCATAATCT	CTGGGCTTCTTTCACTCGTC	(GA)22	55°C
RM517	3	30.3	GGCTTACTGGCTTCGATTTG	CGTCTCCTTTGGTTAGTGCC	(CT)15	55°C
RM527	6	61.2	GGCTCGATCTAGAAAATCCG	TTGCACAGGTTGCGATAGAG	(GA)17	55°C
RM3	6	92.4	ACACTGTAGCGGCCACTG	CCTCCACTGCTCCACATCTT	(GA)2GG(GA)25	55°C
RM337	8	0.1	GTAGGAAAGGAAGGGCAGAG	CGATAGATAGCTAGATGTGGCC	(CTT)4-19-(CTT)8	55°C
RM72	8	60.9	CCGGCGATAAAACAATGAG	GCATCGGTCCTAACTAAGGG	(TAT)5C(ATT)15	55°C
RM316	9	1.8	CTAGTTGGGCATACGATGGC	ACGCTTATATGTTACGTCAAC	(GT)8-(TG)9 (TTTG)4 (TG)4	55°C
RM257	9	79.7	CAGTTCCGAGCAAGAGTACTC	GGATCGGACGTGGCATATG	(CT)24	55°C
RM271	10	59.4	TCAGATCTACAATTCCATCC	TCGGTGAGACCTAGAGAGCC	(GA)15	55°C
RM171	10	92.8	AACGCGAGGACACGTACTTAC	ACGAGATACGTACGCCTTTG	(GATG)5	55°C
RM484	10	102.9	TCTCCCTCCTCACCATTGTC	TGCTGCCCTCTCTCTCTCTC	(AT)9	55°C
RM20A	12	0	ATCTTGTCCCTGCAGGTCAT	GAAACAGAGGCACATTTCATTG	(ATT)14	55°C
RM511	12	59.8	CTTCGATCCGGT GACGAC	AACGAAAGCGAAGCTGTCTC	(GAC)7	55°C
RM309	12	74.5	GTAGATCACGCACCTTTCTGG	AGAAGGCCTCCGGTGAAG	(GT)13	55°C
RM519	12	94.8	AGAGAGCCCCTAAATTTCCG	AGGTACGCTCACCTGTGGAC	(AAG)8	55°C

#### Bulked segregant analysis

Bulked segregant analysis (BSA) was used to detect the major QTL linked to the trait of interest [6]. Two extreme phenotype groups for tolerance each with 10 RILs were constituted to detect the molecular variation using polymorphic SSR markers. Detected polymorphic markers through BSA analysis and their relation with phenotypic effects were further analyzed through molecular mapping method using ICIM v4.0 software.

#### Statistical analysis

Analysis of mean, range, skewness and kurtosis of all 190 recombinant lines and their parents were estimated from the two year data *i*.*e*. wet seasons, 2014 and 2015. These frequency distributions and main effect of the RILs for various traits were performed by using software SPSS v20.0 [67]. INDOSTAT software [68] was used to determine the correlation coefficients of the studied traits using the RILs. Coefficient of variance (CV) and LSD_5%_ were obtained by using CROPSTAT v7.0 software.

#### Linkage map and QTL analysis

Phenotyping data of eleven morho-physiologic traits *viz*., plant height, leaf rolling, leaf drying, panicle length, percentage of panicle emergence, harvest index, 1000-seed weight, percentage of spikelet fertility, relative water content, cell membrane stability and grain yield along with genotypic data of 190 RILs and parents were utilized for construction of linkage map by using the software inclusive composite interval mapping v4.0 (ICIM v4.0) [67]. Analysis of composite interval mapping (CIM) and additive effect in relation to QTL mapping were used to calculate the association of phenotypic and molecular proportions for the development of linkage map. The walking speed along chromosomes for all QTLs was 1.0cM, and threshold value of LOD of > 2.5 with 1000 permutations for P<0.05 were considered. The QTLs were named according to the nomenclatural guidelines given in [69].

## Results

### Variation in morpho-physiological traits of RILs under reproductive stage drought stress

The recombinant inbred lines and parents were phenotyped for eleven morpho-physiological traits under reproductive stage drought stress during wet seasons, 2014 and 2015. All the mopho-physiological traits estimated from the contrasting parents showed significant variations between the parents (Table 3). The morpho-physiological parameters estimates *viz*., percentage of panicle emergence, harvest index, 1000-grain weight, percentage of spikelet fertility, relative water content, cell membrane stability and grain yield were observed to be high in tolerant parent, CR 143-2-2 than susceptible parent Krishnahamsa except plant height, leaf rolling, leaf drying and panicle length. Thus, the selection of contrasting parents for development of mapping population for mapping of these traits was effective.

**Table 3:** Mean estimates of morpho-physiological traits in contrasting parents (CR143-2-2 and Krishnahamsa) under reproductive stage drought stress.

Sl. No.	Phenotyping traits	CR 143-2-2 (Tolerant Parent)	Krishnahamsa (Susceptible Parent)
1	Plant height (cm)	66	74
2	Leaf rolling (SES score)	0.5	6.0
3	Leaf drying (SES score)	0.5	5.5
4	Panicle length (cm)	18.81	20.94
5	Panicle emergence (%)	95	93
6	Harvest index	0.48	0.234
7	1000- seed weight (g)	22.94	20.25
8	Spikelet fertility (%)	84.62	55.10
9	Relative water content (%)	90.57	56.78
10	Cell membrane stability (%)	86.73	51.14
11	Grain yield (g)	5.82	1.23

Plant height (PH) is an important parameter controlling plant type of the RILs under stress condition. In our study, significant variation was obtained among the recombinant lines for this trait (Table 4). Plant height of the studied RILs showed high variation ranging from 45.67cm to 117.92cm. Leaf rolling (LR) and leaf drying (LD) are the two useful traits for identifying drought donor parents. The tolerant donor, CR 143-2-2 showed very low SES score compared to the susceptible parent, Krishnahamsa (Table 3). In this investigation, a wide variation in LR was observed showing a range of 0 to 9.0 with mean of 2.8 among the RILs. Similarly, LD also showed a wide variation with mean value of 1.57 among the RILs under drought stress condition (Table 4). Coefficient of variation and LSD_5%_ for LR were 12.5 and 0.95 while the values for LD were 14.1 and 2.0, respectively. A very high heritability of 91 and 92% were computed for both LR and LD traits, respectively.

**Table 4:** Mean statistical parameters estimated from RILs for eleven morpho-physiological traits under reproductive stage drought stress.

Traits	Mean	Range	Skewness	Kurtosis	CV (%)	LSD_(5%)_
PH (cm)	83.96	45.67–117.92	0.008	0.16	6.3	13.79
LR	2.8	0–9.0	0.92	0.12	12.5	0.95
LD	1.57	0–7.0	1.80	2.43	14.1	0.62
PL (cm)	20.12	14.38–25.75	0.014	0.005	9.8	3.89
PE (%)	95.63	80–100	-1.25	3.55	3.7	6.98
HI (%)	0.4	0.152–0.583	-0.23	0.71	15.4	0.08
TSW(g)	20.25	5.51–33.22	-0.8	1.98	6.0	2.38
SF (%)	74.02	44.68–91.96	-0.78	0.24	16.8	18.48
RWC (%)	75.52	12.08–98.16	0.18	1.18	5.9	8.74
CMS	63.42	4.29–91.52	-0.78	0.4	10.0	12.54
YLD (g)	3.75	0.82–7.68	0.79	1.38	17	0.92

PH = plant height, LR = leaf rolling, LD = leaf drying, PL = panicle length, PE = % of panicle emergence, HI = harvest index, TSW = thousand seed weight, SF = spikelet fertility, RWC = relative water content, CMS = cell membrane stability, YLD = grain yield, CV = coefficient of variation, LSD = least square deviation, PCV = phenotypic coefficient of variation, GCV = genotypic coefficient of variation, H^2^ = heritability (broad sense)

The traits, panicle length (PL) and panicle emergence (PE) showed variations in both the parents and among the RILs. Panicle length had a range of 14.38 to 25.75cm, whereas panicle emergence showed 80–100% among RILs. Under the stress, both the parents showed a clear variation for the trait harvest index (HI) and grain yield (YLD). CR 143-2-2 showed higher value of HI (0.48) compared to the susceptible parent, Krishnahamsa showing value of 0.23. Single plant yield (YLD) obtained from CR 143-2-2 (5.82g) was much higher compared to Krishnahamsa (1.23g). It varied from 0.82 to 5.68 with mean of 3.75 for the RILs under the stress. Harvest index ranged from 0.152 to 0.583 showing a mean of 0.4. Coefficient of variation and LSD_5%_ for HI were 15.4 and 0.12 while the values for YLD were 22.5 and 1.22, respectively.

A wide variation for 1000-seed weight (TSW), spikelet fertility (SF) and relative water content (RWC) was observed in both the parents, CR143-2-2 and Krishnahamsa (Table 3). The range of TSW, SF and RWC in the RILs also showed variation of 5.51–33.22, 44.68–91.96 and 12.08–98.16 with a mean of 20.25, 74.02 and 75.52, respectively. High heritability (broad sense) values of 92 and 93% were obtained for TSW and RWC, respectively. The variation for cell membrane stability (CMS) was also higher.

### Frequency distributions

**Fig 1:**
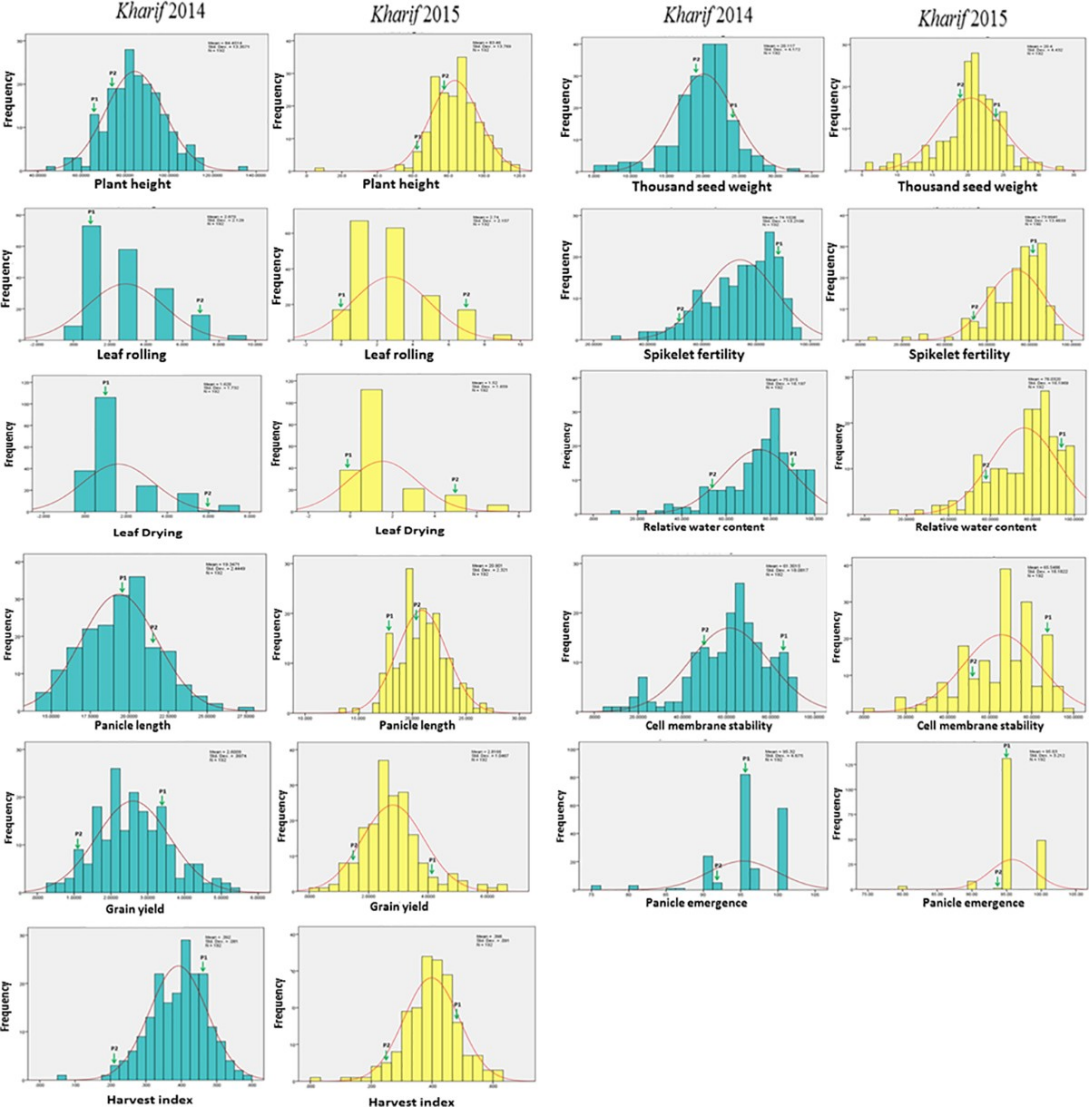
Frequency histogram and phenotypic distribution curves of eleven morpho-physiological traits obtained from the RILs of CR 143-2-2 / Krishnahamsa.

The frequency distributions of 190 RILs along with parents for all the eleven morpho-physiological traits are depicted in the Fig 1. Wide variation was observed for each trait in the parental lines depicted as P1 (tolerant parent) and P2 (susceptible parent). Skewness and kurtosis values of the respective identified traits were estimated and are provided in Table 4. With both the positive values of skewness and kurtosis, six morpho-physiological traits *viz*., plant height, leaf rolling, leaf drying, panicle length, relative water content and grain yield/plant showed a positive leptokurtic skewed distribution. However, rest of the five traits *viz*., panicle emergence, harvest index, 1000-seed weight, spikelet fertility and cell membrane stability exhibited a positive kurtosis and negative skewness values showing leptokurtic skewed distribution. The skewness and kurtosis for majority of the traits were within the value of a normal distribution curve (Table 4; Fig 1). The deviations in leaf drying, leaf rolling and panicle emergence may be due to behavioral pattern of plant might under the drought condition.

### Correlation of morpho-physiological traits with grain yield/plant under stress

The correlation coefficients of studied morpho-physiological traits among themselves and with grain yield under the stress were observed to be significant (Table 5). Out of the total 55 correlations, 25 correlations were significant at the level of 0.01 and 5 correlations were significant at the level of 0.05. A positive high correlation value was observed for HI and grain yield (r = 0.978**) followed by leaf rolling and leaf drying (Table 5). Also, a negative significant correlation was observed between leaf drying and harvest index (r = -0.203**) at 0.01 level of probability. Under stress condition, grain yield showed significant positive correlation with panicle length, plant height, panicle emergence, harvest index, 1000-seed weight, spikelet fertility and relative water content (Table 5).

**Table 5:** Correlation coefficients of morpho-physiological traits among themselves and with grain yield under reproductive stage drought stress.

Correlation coefficients											
	PH	LR	LD	PL	PE	HI	TSW	SF	RWC	CMS	YLD
**PH**	1	-.016	-.055	.334**	.353**	.233**	.265**	.146*	-.110	-.006	.209**
**LR**	-.016	1	.865**	.106	-.169*	-.197**	-.039	-.349**	.043	-.012	-.197**
**LD**	-.055	.865**	1	.023	-.147*	-.203**	-.121	-.306**	.085	.050	-.191**
**PL**	.334**	.106	.023	1	.030	.236**	.101	-.031	-.196**	-.189**	.252**
**PE**	.353**	-.169*	-.147*	.030	1	.219**	-.035	.356**	-.096	-.071	.201**
**HI**	.233**	-.197**	-.203**	.236**	.219**	1	.314**	.233**	-.025	-.128	.978**
**TSW**	.265**	-.039	-.121	.101	-.035	.314**	1	.108	-.060	-.150*	.302**
**SF**	.146*	-.349**	-.306**	-.031	.356**	.233**	.108	1	.013	-.052	.214**
**RWC**	-.110	.043	.085	-.196**	-.096	-.025	-.060	.013	1	.446**	.311**
**CMS**	-.006	-.012	.050	-.189**	-.071	-.128	-.150*	-.052	.446**	1	-.130
**YLD**	.209**	-.197**	-.191**	.252**	.201**	.978**	.302**	.214**	.311**	-.130	1

** Correlation is significant at the 0.01 level (2-tailed)

* Correlation is significant at the 0.05 level (2-tailed)

PH = plant height, LR = leaf rolling, LD = leaf drying, PL = panicle length, PE = % of panicle emergence, HI = harvest index, TSW = thousand seed weight, SF = spikelet fertility, RWC = relative water content, CMS = cell membrane stability, YLD = grain yield

### QTL mapping of morpho-physiological traits under reproductive stage drought stress

**Fig 2:**
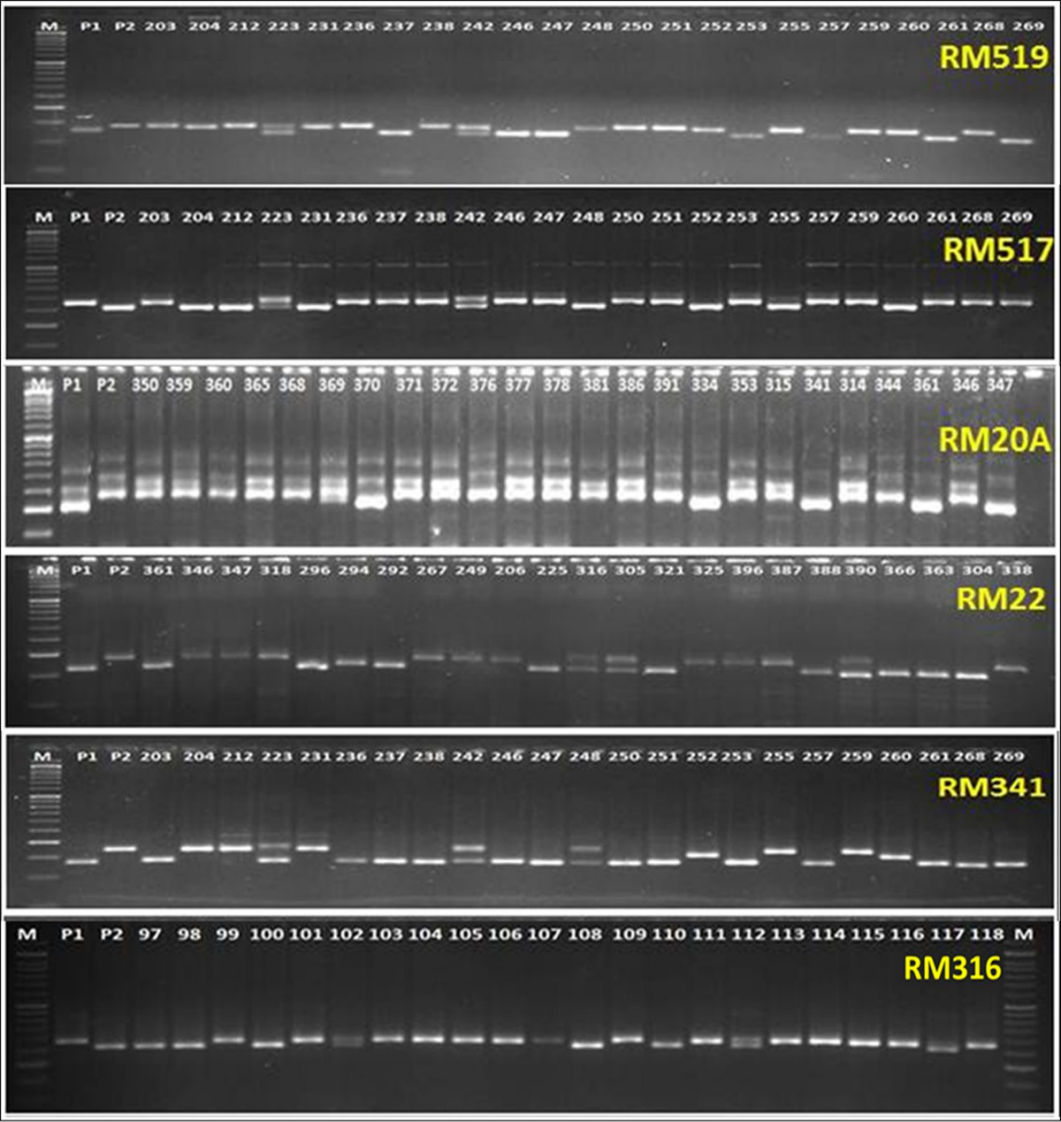
Electrophoregram showing polymorphism pattern of SSR primers with different recombinant lines. The numbers represent the different RI line numbers used in mapping. Respective primer names are given in the right top corner position in each gel photos. **P1:** Tolerant parent; **P2:** Susceptible parent, **M:** 50bp DNA ladder.

In the present investigation, 401 SSR primers were used for detection of parental polymorphism (Table 1). Out of 401 primers, 77 primers were detected to be polymorphic between the parents. Two different extreme RILs bulks (tolerant: B1 and susceptible: B2) were developed and genotyped using the 77 polymorphic primers (Table 2; Fig 2).

**Fig 3:**
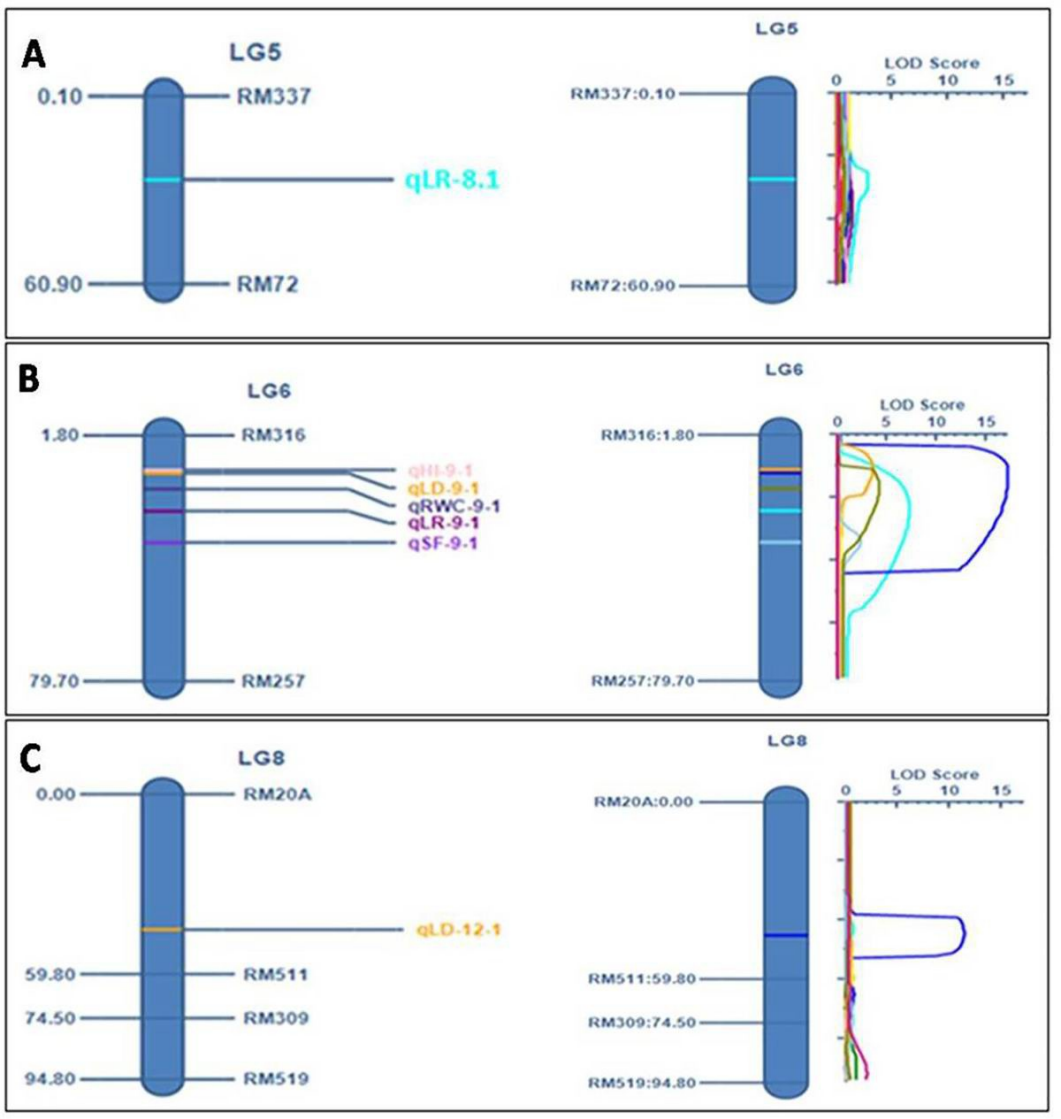
(A) **QTL detected on chromosome 8 (LG5) beyond threshold LOD (2.5)** (light green color represents QTL detected for leaf rolling) (B) **QTL detected on chromosome 9 (LG6) beyond threshold LOD (2.5)** (pink color represents QTL detected for harvest Index, yellow color represents QTL detected for leaf drying, deep blue represents QTL detected for relative water content, maroon color represents QTL detected for leaf rolling and violet color represents QTL detected for spikelet fertility) (C) **QTL detected on chromosome 12 (LG8) beyond threshold LOD (2.5)** (yellow color represents QTL detected for leaf drying).

The analysis using inclusive composite interval mapping (ICIM) method detected seven QTLs linked to leaf drying, leaf rolling, harvest index, spikelet fertility and relative water content traits on three different chromosomes (Chromosome 8, 9 and 12) based on year I phenotyping results (wet season, 2014). However, in year II (wet season, 2015), five QTLs found to be linked to same traits on same chromosome under the stress condition (Table 6; Fig 3B). These seven QTLs (LOD ≥ 2.5) represented five different morpho-physiological traits located on three different chromosomes.

**Table 6:** QTLs detected by linkage analysis using inclusive composite interval mapping.

Year	QTL detected	Chrom#	Position	LM	RM	LOD	PVE(%)	Additive effect
Kharif,2014	q*LR*_8.1_	8	27.1	RM337	RM72	2.78	60.74	-1.68
Kharif,2015	-	-	**-**	**-**	**-**	**-**	**-**	**-**
Pooled	-	-	**-**	**-**	**-**	**-**	**-**	**-**
Kharif,2014	q*LR*_9.1_	9	25.8	RM316	RM257	7.20	67.46	-1.90
Kharif,2015	q*LR*_9.1_	9	19.8	RM316	RM257	5.41	59.03	-1.80
Pooled	q*LR*_9.1_	9	22.8	RM316	RM257	8.21	65.99	-1.82
Kharif,2014	q*LD*_9.1_	9	13.8	RM316	RM257	17.18	70.34	-1.94
Kharif,2015	q*LD*_9.1_	9	15.8	RM316	RM257	21.85	77.86	-1.64
Pooled	q*LD*_9.1_	9	14.8	RM316	RM257	27.85	80.18	-1.81
Kharif,2014	q*LD*_12.1_	12	45.0	RM20A	RM511	11.35	71.72	1.82
Kharif,2015	-	-	-	-	-	-	-	-
Pooled	-	-	-	-	-	-	-	-
Kharif,2014	q*HI*_9.1_	9	12.8	RM316	RM257	3.51	52.40	0.01
Kharif,2015	q*HI*_9.1_	9	12.8	RM316	RM257	7.02	60.20	0.01
Pooled	q*HI*_9.1_	9	11.8	RM316	RM257	5.21	56.45	0.01
Kharif,2014	q*SF*_9.1_	9	35.8	RM316	RM257	2.26	49.85	9.88
Kharif,2015	q*SF*_9.1_	9	12.8	RM316	RM257	3.50	45.79	11.68
Pooled	q*SF*_9.1_	9	23.8	RM316	RM257	3.58	54.5	8.35
Kharif,2014	q*RWC*_9.1_	9	18.8	RM316	RM257	4.13	59.62	15.14
Kharif,2015	q*RWC*_9.1_	9	23.8	RM316	RM257	4.78	62.65	14.52
Pooled	q*RWC*_9.1_	9	21.8	RM316	RM257	4.27	60.87	14.56

LR = leaf rolling, LD = leaf drying, HI = harvest index, SF = spikelet fertility, RWC = relative water content, LM = left marker, RM = right marker

The QTL for leaf rolling was detected on both chromosome 8 and 9, the consistent QTL was detected on chromosome 9 (Fig 3A and 3B). The detected linkage of QTL, *qLR*_*9*.*1*_ controlling leaf rolling character was located on chromosome 9 and observed consistently in both the years in the same physical position on the chromosome under the reproductive stage drought stress (Fig 3B; Table 6). LOD value of 8.21 and phenotypic variance of 65.99 were obtained for the trait from the pooled data analysis (Fig 3B; Table 6). A distinct peak with additive effect of -1.82 was observed in the graphical representation of QTL analysis for *qLR*_*9*.1_ in the linkage map (Fig 3B). Position of *qLR*_*9*.1_ was at 22.8cM within the marker interval of RM316 and RM257 on chromosome 9 (Table 6). In case of *qLR*_*8*.1,_ the QTL was detected on chromosome 8 within the marker interval of RM337-RM72 and found only in a single experimental year, 2014 (Fig 3A). The LOD and PVE (%) of *qLR*_*8*.1_ was 2.78 and 60.74, respectively (Table 6).

The QTL for leaf drying was detected on chromosome 9 and 12 (Fig 3B). However, the QTL on chromosome 9 was found to be consistent and detected in both the years on the same position (Table 6). The analysis for leaf drying showed a QTL, *qLD*_*9*.1_ controlling the character was on chromosome 9 under stress tolerance in both the years (Fig 3B). A high LOD value of 27.85 and phenotypic variance of 80.18 were obtained for the QTL, *qLD9*.*1* from the pooled data analysis (Fig 3B; Table 6). A distinct peak was observed in the graphical representation of QTL analysis for *qLD*_*9*.1_ in the linkage map with additive effect of -1.81 (Fig 3B). Position of *qLD*_*9*.1_ was at 14.8cM within the marker interval of RM316 and RM257 on chromosome 9 (Table 6). Another QTL for the trait, *qLD*_*12*.1_ was also detected on chromosome 12 within the marker interval of RM20A-RM511 based on a single experiment year I results (Fig 3C). The LOD and PVE (%) of *qLD*_*12*.1_ were 11.35 and 71.72, respectively (Table 6).

The QTL, *qHI*_*9*.1_ located on chromosome 9 at 11.8cM position within the marker interval of RM316 and RM257 was detected to be linked to the trait harvest index under the stress (Fig 3B; Table 6). Additive effect obtained from the study was 0.01 which was minimal effect in this QTL mapping (Fig 3B). The linkage was detected with LOD value of 5.21 showing phenotypic variance % of 56.45 for the trait, harvest index (Fig 3B). Analysis using the software detected the QTL linked to the trait in both experiment years (2014 and 2015). The location on chromosome 9 was same in both the years’ phenotypic data of *qHI*_*9*.1_ (Table 6). In addition, *qSF*_*9*.1_ controlling spikelet fertility was detected as an effective QTL showing LOD value of 3.58 with PVE% of 54.5 (Fig 3B; Table 6). A clear peak was found in the linkage group at 23.8cM position within the maker interval of RM316-RM257 (Fig 3B). QTL for relative water content (*qRWC*_*9*.*1*_) was found at the position of 18.8cM on the basis of wet season 2014 RWC phenotyping data. A significant peak with LOD of 4.13 and PVE% of 59.62 were observed in the marker interval of RM316 and RM257 (Fig 3B; Table 6). On the basis of wet season, 2015 RWC data, the QTL (*qRWC*_*9*.*1*_) was again detected within the same marker interval but at a distance of 23.8cM on the chromosome. The LOD value and PVE (%) were found to be 4.78 and 62.65%, respectively in the second year (Fig 3B; Table 6). It was estimated to be present on chromosome 9 at 21.8cM with LOD value and PVE (%) of 4.27 and 60.87, respectively within the same marker interval based on pooled data analysis. The additive effect of the QTL was found to be 14.56.

## Discussion

Though yield QTLs under reproductive stage drought tolerance are previously reported, but the information on morpho-physiological traits linked to molecular markers under reproductive stress are very limited. The present results revealed the marker information on the linkage of various markers with key morpho-physiological traits under the stress situation. The statistical parameters estimated for morpho-physiological from the RILs showed significant correlations values among themselves under the stress. A continuous frequency distribution curve for plant height, panicle length, harvest index, grain yield and cell membrane stability was observed from the distribution curve (Fig 1). This indicated the effectiveness of the mapping population and selection of contrasting parents for detection of various QTLs controlling various morpho-physiological traits during reproductive stage drought stress.

Out of the eleven physiological traits studied, five traits *viz*., leaf rolling (*qLR*_*9*.*1*_), leaf drying (*qLD*_*9*.*1*_), harvest index (*qHI*_*9*.*1*_), spikelet fertility (*qSF*_*9*.*1*_) and relative water content (*qRWC*_*9*.*1)*_ were tagged by QTL composite interval mapping. Chromosome 9 is found to be the most important chromosome showing linkage with five different morpho-physiological traits. Basing on the phenotyping data of both the years, QTLs namely *qLR*_*9*.*1*_, *qLD*_*9*.*1*_, *qHI*_*9*.*1*_, *qSF*_*9*.*1*_ and *qRWC*_*9*.*1*_ were observed within the marker interval of RM316 and RM257 on chromosome 9. These QTLs were found to be consistent over both the years. Hence, these QTLs can be useful in marker-assisted breeding for improvement of drought tolerance in rice.

Delaying in leaf drying and leaf rolling are the two required traits for improvement of drought tolerance in rice. Under extreme stress condition, scores for leaf drying generated at all growth stages of rice for determining drought tolerance need to be in moderate to high score. Hence, these consistent QTLs detected from the mapping study should be pyramided in the recurrent parent for improvement of drought tolerance for reproductive stage stress tolerance in rice. Previous mapping results of leaf rolling under drought by [46] had suggested four QTLs for drought sensitivity index detected, *lr*_*8*.*1*_, *lr*_*4*.*1*_, *lr*_*10*.*1*_, and *lr*_*12*.*1*_. Also, earlier finding suggests that QTL (*lr*_*8*.*1*_) for relative water content and leaf rolling was already mapped at equivalent regions (near RM72) on chromosome 8 [43]. Experimental findings of [48] suggested 3 markers (RM212, RM302 and RM3825) linked to morphological trait, leaf rolling on chromosome 1 under the drought stress condition. These three markers were also linked to leaf drying under drought stress. As per findings of [45], 5 QTLs were detected for leaf rolling trait amongst which one QTL was on chromosome 9 at a distance of 65.6cM. Also in another experiment conducted under drought stress condition, phenotypic variance of 24.8% was detected for the leaf rolling QTL on chromosome 6 [50]. Another QTL for the trait also reported on chromosome 12 near RM101. However, in our study, we mapped QTL for leaf rolling on chromosome 9 at a position of 22.8cM with LOD of 8.21, PVE (%) of 65.99 with additive effect of -1.82. The distinct location detected and the phenotypic variance contribution of the trait suggest this QTL as a novel QTL controlling leaf rolling during reproductive stage drought tolerance in rice. Our mapping results detected another QTL for leaf rolling trait, located in the marker interval of RM72 and RM337 at a distance of 27.1cM on chromosome 8 (Fig 3B). This QTL has already been reported by [46] and [43]. Hence, this QTL is validated and can be useful for marker-assisted breeding.

Previous mapping results indicate that RM8085 at 139.9cM linked to leaf drying and leaf rolling traits on chromosome 1 under severe drought stress condition [49]. Published results of [45] detected four QTLs for leaf drying, one each on chromosome 1, 3, 3 and 11, respectively. Mapping results of [47] also suggests that *qld*_*1*.*1*_ located on chromosome 1 controls the leaf drying under drought stress. However, the current finding on mapping of leaf drying is detected within the marker interval of RM316-RM257 on chromosome 9 with LOD value 27.85, PVE (%) 80.18 and additive effect of -1.81. Therefore, this is a different from the earlier mapping results for the trait. Thus, this may be a new QTL, controlling leaf drying under drought stress and designated as *qLD*_*9*.*1*_. Our mapping results showed another leaf drying linked QTL, *qLD*_*12*.*1*_ *which* is located within the boundary of RM20A and RM511 at a distance of 45cM on chromosome 12 (Fig 3C). As no QTL is reported in this region of the chromosome from earlier study, the detected QTL, *qLD*_*12*.*1*_ controlling leaf drying under drought stress showing a percentage of phenotypic variance 71.72 may be a novel QTL for the trait.

Consistent QTLs for harvest index, straw yield, and grain yield under drought stress were reported earlier for RM314 on chromosome 6 with the experiments regulated for several seasons [50]. Earlier study on mapping for HI suggested the linkage of the trait on chromosome 1 detected a QTL with RM315 under stress situation [42]. Another QTL on chromosome 1 designated as *qhi*_*1*.*1*,_ reported earlier showed expression only under extreme drought situation [26]. A QTL for harvest index on chromosome 2 was detected with 65.6% phenotypic variance under the stress [47]. As per their reports, the QTLs for epicuticular wax, harvest index, and water loss rate from processed cut leaves under stress were detected to be co-located with QTLs related to root and shoot related drought tolerance characters in particular rice progenies and might be functional for rain-fed rice development. As under drought situation, the correlation among grain yield (GY) and harvest index (HI) showed high value specifying that HI is a principal component of grain yield under this stress. Therefore, genetic enhancement of HI would require for increasing GY [70–71]. The QTLs on chromosome 3 classified as *qhi*_*3*.*4*_, *qhi*_*3*.*2*_, *qhi*_*3*.*1*_, *qhi*_*3*.*3*_, and *qhi*_*3*.*5*_ were reported in the marker interval of RG104-RM231 [26]. Another QTL reported on chromosome 1 specified as *qhi*_*1*.*1*_ that expresses only under severe stress condition [26]. However, the QTL detected in our experiment for controlling HI under drought stress was located on chromosome 9 within the marker interval of RM316-RM267 showing LOD of 5.21 with PVE (%) of 56.45 and additive effect of 0.01 was clearly different than the earlier mapping reports for the trait. Therefore, the QTL, *qHI*_*9*.*1*_ detected by us under the reproductive stage drought stress tolerance may be a novel QTL.

Grain yield showed positive correlations with spikelet fertility under reproductive stage drought stress. When spikelet fertility decreased, grain yield also consequently decreased. Pollen formation in rice plants is highly sensitive to drought stress. Abiotic stress during meiotic stage results pollination failure, pollen sterility leading to zygotic abortion and finally spikelet death, but female fertility is affected only under extreme stress. Previous publication on three QTLs, designated as *qpss*_*5*.*1*_, *qpss*_*4*.*1*_, and *qpss*_*9*.*1*_ reported on chromosomes 5, 4, and 9 were under the irrigated situations [26]. Under slight water stress condition, the QTLs *qpss*_*5*.*3*_ and *qpss*_*5*.*2*_ were also on chromosome 5 reported. The QTLs responsible for *qpss*_*5*.*3*_ and *qpss*_*5*.*2*_ were observed in the same marker interval of EM15_4 to CDO20, as *qpss*_*5*.*1*._ Under more water stress level, the QTLs like *qpss*_*8*.*2*_ and *qpss*_*8*.*1*_ were tagged. The *qpss*_*8*.*2*_ and *qpss*_*8*.*1*_ were both detected in the marker interval of ME5_4 to RM256. However, the QTL detected by us, *q*SF_9.1_ under water stress is nearer to the location of *qpss*_*9*.*1*_ reported by [26]. The peak marker RM219 for *qpss*_*9*.*1*_ was on 11.7cM position which is not far from our detected QTL, *qSF*_*9*.*1*_ located at 12.8cM position in the same chromosome. A QTL was reported on chromosome 9 for spikelet sterility [72]. However, this quantitative trait locus was located at different position of the reported QTL, *qpss*_*9*.*1*_. The two QTLs, *qpss*_*8*.*2*_ and *qpss*_*8*.*1*_, which showed its effect under moderate and extreme drought stress, were also linked with OA (*oa*_*8*.*1*_) QTL at the same position as per report of [72]. While [40] detected QTL for OA, at RG1-RM80 marker interval. Mapping results of [27] showed four QTLs namely *qRsf3*, *qRsf5*, *qRsf8* and *qRsf9* controlling spikelet fertility under drought conditions. Therefore, the result concluded that our detected QTL, *q*SF_9.1_ might be present on the same locus on chromosome 9 related to spikelet fertility as reported by earlier mapping results. Thus, the detected QTL, *qSF9*.*1* by us is validated in this mapping population and hence can be useful in marker-assisted drought improvement breeding in rice.

Under water stress condition, higher value of relative water content (RWC) is obtained from drought tolerant genotypes at reproductive stage compared to susceptible genotypes. Earlier mapping work of [70] indicated that in the marker interval of R2417-RM212-C813 on chromosome 1 associated with RWC under field drought condition. A QTL, *qrwc*_*11*.*1*_ on chromosome 11 was reported earlier in between marker interval of RM254-RG1465 for controlling RWC in rice [47]. [3] reported the physiological response of relative water content by *qtl*_*12*.*1*_ affecting grain yield under drought condition. Earlier mapping results of [43] reported QTLs for RWC on chromosome 1, 3 and 8. Another reports of [73] constructed the linkage map on chromosome 1, 6 and 12 for relative water content trait. The QTL for the trait detected by us was on chromosome 9 in between the marker interval of RM316-RM257 at 21.8cM position. This location on the chromosome 9 for this trait was not mapped earlier as per the mapping reports. Thus, *qRWC*_9.1_ controlling relative water content may be considered as a novel QTL from our mapping results.

We observed a significant correlation of plant height (r = 0.209**), panicle length (r = 0.252**), panicle emergence (r = 0.201**), 1000-seed weight (r = 0.302**) with grain yield. These traits were reported earlier as important drought controlling factors responsible for growth, development and yield parameters in rice [74]. However, there were no QTL found linked to these traits showing above the thresh hold LOD values in our mapping population. Cell membrane stability (CMS) is considered to be one of the major selection indices of drought tolerance in cereals [41]. A negative non-significant correlation coefficient value of -0.134 for CMS was observed with grain yield from this study. But, no QTL showing LOD value more than the threshold level was detected for this trait.

## Conclusion

The present study showed the nature of correlation of morpho-physiological parameters with themselves and with grain yields under reproductive stage drought stress. Five consistent QTLs were detected *viz*., *qLR*_*9*.1,_ *qLD*_*9*.1,_ *qHI*_*9*.1,_ *qSF*_*9*.1,_ and *qRWC*_*9*.1_ controlling leaf rolling, leaf drying, harvest index, spikelet fertility and relative water content, respectively under reproductive stage drought stress. Also, *qLR8*.*1* was validated in this mapping population and useful in marker-assisted breeding programs. The correlated physiological traits may be useful in selecting desirable drought tolerant plants for reproductive stage drought stress. Also these identified QTL regions can be useful in marker-assisted breeding program for development of drought tolerant rice plant.
